# An Effective Hybrid Treatment for Persistent Sciatic Artery Aneurysm

**DOI:** 10.1016/j.ejvsvf.2025.08.002

**Published:** 2025-08-18

**Authors:** Alessandro Ciolli, Francesco Stilo, Vincenzo Cirimele, Nunzio Montelione

**Affiliations:** a Vascular Surgery Unit, Fondazione Policlinico Campus Bio-Medico di Roma, Rome, Italy; b Vascular Surgery Unit, Fondazione Policlinico Universitario Agostino Gemelli di Roma, Rome, Italy; c Radiology Department, Fondazione Policlinico Campus Bio-Medico di Roma, Rome, Italy

**Keywords:** Aneurysmal degeneration, Embolisation, Endovascular, Persistent sciatic artery, Surgical bypass

## Abstract

**Introduction:**

Persistent sciatic artery (PSA) is a rare congenital vascular anomaly that is often asymptomatic; however, it can be associated with aneurysm formation and potential complications. Here, the case of a 61 year old man presenting with bilateral persistent sciatic artery aneurysms (PSAAs) who underwent right sided hybrid treatment for rest pain and aneurysm thrombosis is reported.

**Report:**

Hybrid treatment of the right PSAA was performed by surgical bypass using the *in situ* great saphenous vein, from the common femoral artery to the posterior tibial artery and staged percutaneous aneurysm embolisation with controlled release coils and a vascular plug. The post-operative course was uneventful, and computed tomography angiography at six months confirmed right PSAA exclusion and graft patency. At 12 months follow up, the bypass was patent, and the patient remained free of ischaemic symptoms. The left PSAA remained asymptomatic.

**Discussion:**

PSAAs can be associated with chronic limb threatening ischaemia due to complications such as thrombosis, and a hybrid approach can treat this successfully.

## INTRODUCTION

Persistent sciatic artery (PSA) is a rare vascular abnormality with an incidence of 0.01–0.05%. It is often asymptomatic but prone to aneurysm formation (persistent sciatic artery aneurysm [PSAA]) in 15–44% of cases.^1^^,^^2^ PSAA may lead to severe complications, including chronic limb threatening ischaemia, acute limb ischaemia due to thromboembolism, aneurysm rupture, and significant pain due to sciatic nerve compression.^3^ This article presents the case of a patient with bilateral PSAAs and right sided chronic limb threatening ischaemia successfully treated using a staged approach with below knee surgical bypass using a venous conduit followed by percutaneous PSAA embolisation.

## REPORT

A 61 year old man with hypertension, dyslipidaemia, and hypertensive heart disease presented with a one month history of right lower limb rest pain. Physical examination showed palpable femoral pulses bilaterally but absent peripheral pulses, with no tissue loss, gangrene, or pulsatile mass. Transcutaneous oxygen tension levels were 33 mmHg in the right lower limb and 52 mmHg in the left lower limb. Duplex ultrasound (DUS) revealed bilateral normal flow in the common, deep femoral, and proximal portion of the superficial femoral arteries; conversely, popliteal multilevel crural occlusions with monophasic flow, particularly in the right posterior tibial artery (PTA) and all crural vessels of the left lower leg, were noted. Venous mapping confirmed a suitable greater saphenous vein (GSV).

**Figure 1:**
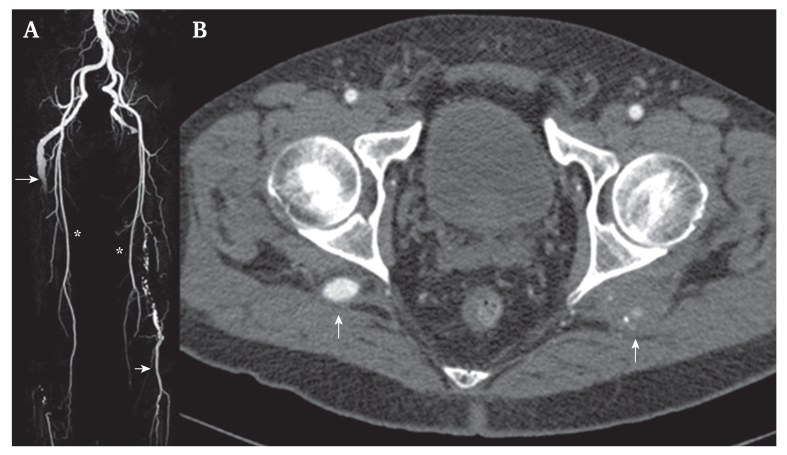
(A) Coronal maximum intensity projection and (B) axial computed tomography images showed persistent bilateral sciatic artery (arrows) affected by occluded aneurysmal dilatations, small calibre bilateral superficial femoral arteries (stars), and patent left side popliteal and tibial vessels (arrow).

Computed tomography angiography (CTA) demonstrated bilateral PSAAs (right: 3.3 cm; left: 2.5 cm) (Fig. 1). In the right lower limb, CTA confirmed complete persistent sciatic artery (PSA) with a small-caliber (<4 mm) superficial femoral artery, occlusion of the popliteal artery and tibioperoneal trunk, and a patent posterior tibial artery (PTA). The left PSAA was occluded with a small calibre (<4 mm) superficial femoral artery and a patent popliteal artery in continuity with the three tibial vessels, showing no distal embolisation, suggesting conservative management. Considering the ischaemic symptoms in the right limb, a revascularisation strategy was planned. Firstly, a surgical bypass from the common femoral artery to the PTA was performed using *in situ* GSV under general anaesthesia. A medial skin incision was made along the course of the GSV to isolate the vein and ligate all its branches. The valve lysis was carried out using the Chevalier Valvulotome (LeMaitre Vascular, Inc., Burlington, MA, USA). Intra-operative DUS examination was used to ensure the complete ligation of any remaining collaterals and to verify the flow along the graft distal to the target vessel.

**Figure 2:**
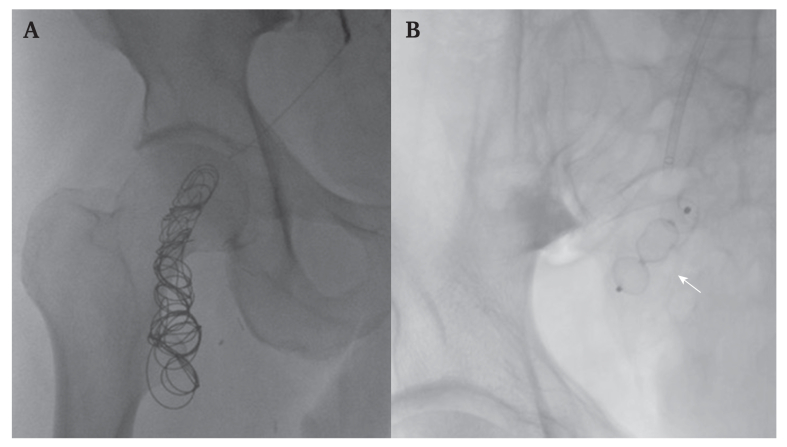
(A) Fluoroscopic images indicating coils (two controlled release coils 20 × 60 mm and 18 × 57 mm; Penumbra, Inc., Alameda, CA, USA) and (B) the Amplatzer Vascular Plug II (Abbott Laboratories, Chicago, IL, USA) (arrow) used to exclude a persistent right sciatic artery aneurysm.

**Figure 3:**
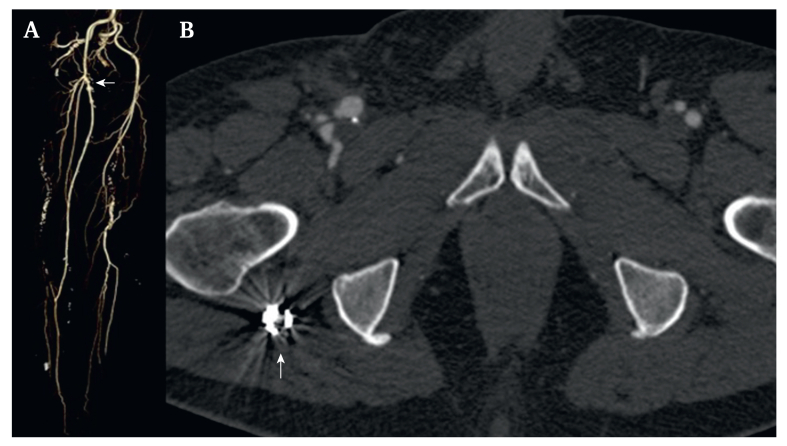
(A) Coronal volume rendering technique and (B) axial computed tomographic images demonstrated the successful exclusion of the persistent sciatic artery aneurysm and patency of the bypass between the common femoral artery (arrow) and the right posterior tibial artery.

The post-operative course was uneventful with mild reperfusion oedema and complete resolution of rest pain. The patient was discharged on post-operative day six with instruction to take aspirin 100 mg, rosuvastatin 10 mg daily, and rivaroxaban 2.5 mg twice daily. Three weeks later, ultrasound guided percutaneous access was successfully obtained via the left common femoral artery under local anaesthesia. A 45 cm 6 F Flexor sheath (Cook Medical Inc., Bloomington, IN, USA) was advanced across the aortic bifurcation to selectively catheterise the internal iliac artery. Angiography confirmed a partially thrombosed PSAA. Using a Carnelian 2.7 microcatheter (Tokai Medical Products, Aichi, Japan), embolisation of the PSA was performed using two controlled release coils (20 × 60 mm and 18 × 57 mm; Penumbra, Inc., Alameda, CA, USA) and an Amplatzer Vascular Plug II (12 × 9 mm; Abbott Laboratories, Chicago, IL, USA). These devices were deployed just distal to the gluteal artery origin, achieving complete aneurysm exclusion while preserving the patency of the internal iliac artery and its branches (Fig. 2), as confirmed by post-operative CTA (Fig. 3).

The post-operative course was uneventful, and the patient was discharged on the third post-operative day. Follow up was planned through clinical evaluation and DUS examination at one, three, six, and 12 months. At 12 months follow up, the patient was in good clinical condition; DUS revealed bypass patency and a well perfused PTA, and transcutaneous oxygen tension was 62 mmHg. The left PSAA is currently asymptomatic, under close follow up, and scheduled for treatment in the event of aneurysm enlargement or symptoms.

## DISCUSSION

During embryonic development, around the fourth to fifth week of gestation, the sciatic artery, originating from the umbilical artery, acts as the principal vascular supply to the lower limbs. By the eighth week, it typically regresses in the distal thigh, with its proximal portion contributing to the superior and inferior gluteal arteries and its distal portion forming segments of the popliteal and peroneal arteries. It normally regresses by the 12th week. If the femoral artery fails to develop, the sciatic artery may persist as the dominant vessel supplying blood to the thigh, leading to the presence of a PSA. Bilateral PSAs account for up to 30% of all cases, affecting both sexes equally.

PSA was first documented by Green in 1832, followed by angiographic studies conducted by Cowie in 1960. Over the decades, several anatomical classifications of PSA have been proposed,^4^^,^^5^ with the most recent, introduced by Ahn *et al.*^6^ in 2016, offering a refined system that incorporates the presence of aneurysms and the development of the PSA and superficial femoral artery. Among diagnostic imaging modalities, CTA is effective in assessing anatomic details, aneurysm size, and the status of adjacent structures.^7^

To prevent complications, PSA treatment depends on the patient's symptoms, anatomic classification, and comorbidities. In asymptomatic patients with PSA without aneurysmal degeneration, conservative management with annual surveillance, including physical examination and imaging studies, is suggested. Symptomatic patients with sciatic nerve injury, thromboembolic events, or PSAA require invasive treatment.^8^

The type of surgery depends on whether the PSA is complete or incomplete. For incomplete PSA, where femoral and collateral arteries support the lower limb vasculature, surgical ligation or embolisation can be performed without ischaemic consequences. For complete PSA, a limb revascularisation strategy is recommended; in the event of complete PSA and aneurysmal degeneration, a hybrid approach, combining surgical and endovascular techniques, can exclude the aneurysm while preserving limb perfusion.^9^

The present case involved a type 2A PSA, as classified by the Pillet-Gauffre classification, corresponding to class IIIa in the Ahn *et al.*^6^ classification, that was effectively managed using a hybrid strategy. Although previous hybrid cases employed prosthetic grafts,^10^ a venous graft was chosen in this case owing to its superior long term patency rates, especially in the setting of a below the knee arterial occlusion.

This approach firstly addressed the ischaemic symptoms and then the aneurysmal aspects, successfully restoring blood flow while avoiding time consuming open surgery in addressing both aspects, representing a low risk hybrid strategy in managing such pathology.

## ETHICAL STATEMENT

The authors adhered to all research ethics guidelines of the discipline; particularly, all procedures performed in studies involving human participants were in accordance with the ethical standards of the institutional and or national research committee and with the 1964 Declaration of Helsinki and its later amendments or comparable ethical standards. The patient provided informed consent at all stages of the study for the publication, reproduction, and use of photographs, recordings, audiovisual material, and textual content related to their case history in any scientific publication, presentation, or promotional material. The patient has waived any claims regarding the use of this material.

## Funding

This research received no specific grant from any funding agency in the public, commercial, or not-for-profit sectors.

## CONFLICT OF INTEREST

None.
